# Ceftaroline Efficacy and Safety in Treatment of Complicated Skin and Soft Tissue Infection: A Systemic Review and Meta-Analysis of Randomized Controlled Trials

**DOI:** 10.3390/jcm8060776

**Published:** 2019-05-31

**Authors:** Shao-Huan Lan, Shen-Peng Chang, Chih-Cheng Lai, Li-Chin Lu, Chien-Ming Chao

**Affiliations:** 1 School of Pharmaceutical Sciences and Medical Technology, Putian University, Putian 351100, China; shawnlan0713@gmail.com; 2 Department of Pharmacy, Chi Mei Medical Center, Liouying 73657, Taiwan; httremoon@ms.szmc.edu.tw; 3 Department of Intensive Care Medicine, Chi Mei Medical Center, Liouying 73657, Taiwan; dtmed141@gmail.com; 4 School of Management, Putian University, Putian 351100, China; jane90467@gmail.com

**Keywords:** ceftaroline, complicated skin and skin structure infection, vancomycin, methicillin-resistant *Staphylococcus aureus*

## Abstract

This study aims to assess the clinical efficacy and safety of ceftaroline for the treatment of complicated skin and skin structure infections (cSSSIs) in adult patients through meta-analysis. PubMed, Embase, ClinicalTrials.gov, and Cochrane databases were searched up to April 2019. Only randomized controlled trials (RCTs) that evaluated ceftaroline and other comparators for treating cSSSIs in adult patients were included. The primary outcome was the clinical cure rate, whereas the secondary outcomes were clinical failure rate, microbiological eradication rate, relapse rate, and risk of an adverse event (AE). Five RCTs were included. Overall, ceftaroline had a clinical cure rate similar to comparators in the treatment of cSSSIs in the modified intent-to-treat population (risk ratio (RR), 1.00; 95% confidence interval (CI), 0.97–1.04; *I*^2^ = 0%) and in the clinically evaluable population (RR, 1.00; 95% CI, 0.97–1.03; *I*^2^ = 0%). In addition, no significant difference was observed between ceftaroline and comparators for the treatment of infection with *Staphylococcus* *aureus* (RR, 1.01; 95% CI, 0.98–1.05; *I*^2^ = 0%), methicillin-resistant *S. aureus* (RR, 0.99; 95% CI, 0.94–1.05; *I*^2^ = 0%), methicillin-susceptible *S. aureus* (RR, 1.01; 95% CI, 0.96–1.06; *I*^2^ = 26%), *Streptococcus* spp. (RR, 1.07; 95% CI, 0.92–1.24; *I*^2^ = 73%), and Gram-negative bacteria (RR, 0.94; 95% CI, 0.83–1.08; *I*^2^ = 0%). Furthermore, ceftaroline had a similar rate of microbiological eradication (92.2% vs. 92.6%, RR, 1.00; 95% CI, 0.97–1.03; *I*^2^ = 9%) and relapse (6.9% vs. 9.1%, RR, 0.48; 95% CI, 0.14–1.74; *I*^2^ = 0%) as comparators. Finally, the risks of treatment-emergent AEs (RR, 0.96; 95% CI, 0.88–1.05; *I*^2^ = 0%), serious AEs (RR, 1.03; 95% CI, 0.63–1.68; *I*^2^ = 0%), and discontinuation of study drug due to an AE (RR, 0.86; 95% CI, 0.50–1.49; *I*^2^ = 34%) did not differ significantly between ceftaroline and comparators. In conclusion, the clinical efficacy of ceftaroline is as high as that of comparators in the treatment of cSSSIs in adult patients, and this antibiotic is well tolerated like the comparators.

## 1. Introduction

Complicated skin and skin structure infection (cSSSI) is a common type of acute bacterial infection requiring hospitalization [1,2,3]. Appropriate antibiotics are essential for successfully treating cSSSIs. However, antibiotic-resistant bacteria in this clinical setting, particularly methicillin-resistant *Staphylococcus aureus* (MRSA) [4], have largely reduced the treatment options of antibiotics. Currently, glycopeptides, such as vancomycin, teicoplanin, daptomycin, and linezolid, are the commonly recommended antibiotics for cSSSIs caused by MRSA [5].

Ceftaroline—a broad-spectrum cephalosporin—was approved in 2010 for the treatment of cSSSIs and bacterial pneumonia in adult patients. In in vitro studies, ceftaroline exhibited potent antibacterial activity against commonly encountered pathogens, such as *Streptococcus* spp. and *Staphylococcus* spp. (including MRSA) [6,7,8,9,10,11]. According to the findings of these studies [8,9,10,11], ceftaroline can cover most common pathogens causing cSSSIs and can be an appropriate antibiotic for the treatment of cSSSIs. Recently, several randomized trials have assessed the clinical efficacy and safety of ceftaroline for treating cSSSIs and pneumonia in adult patients [12,13,14,15,16,17,18,19]. Regarding pneumonia, a recent meta-analysis showed that ceftaroline has a clinical efficacy rate of 81.2% and >70% success rate against pneumonia caused by *S. pneumonia* and *S. aureus*, respectively, including their multidrug-resistant pathogens [20]. However, an updated meta-analysis comparing the efficacy and safety of ceftaroline and other comparators for the treatment of cSSSIs in adult patients is lacking. Therefore, we conducted this meta-analysis to provide evidence on the efficacy and safety of ceftaroline in adult patients with cSSSIs.

## 2. Materials and Methods

### 2.1. Study Search and Selection

All clinical studies were identified through a systematic review of the literature in PubMed, Embase, ClinicalTrials.gov, and Cochrane databases until April 2019 using the following search terms: “ceftaroline”, “complicated skin and skin structure infection”, “complicated skin and soft tissue infection”, “acute bacterial skin and skin structure infection”, and “adult”. Only randomized controlled studies written in English that compared the clinical efficacy and adverse effects of ceftaroline and other comparators in the treatment of cSSSIs among adult patients were included. Two reviewers (Lan and Chang) searched and examined publications independently to avoid bias. Any disagreement was resolved by a third reviewer (Lai). The following data were extracted from all the included studies: year of publication, study design, countries, antibiotic regimens of ceftaroline and comparators, outcomes, and adverse events (AEs). The modified intention-to-treat (MITT) population consisted of all patients in the intention-to-treat (ITT) population who had a confirmed diagnosis in accordance with the study protocol criteria. The clinically MITT (cMITT) population included patients in the MITT population who met the minimal clinical criteria for cSSSI. The clinically evaluable (CE) population included patients from the cMITT population who received a specified amount of study medication, had a clinical response of cure or failure at the test-of-cure visit, and for whom there were no confounding factors that interfered with the assessment of that outcome. The microbiologically evaluable population included patients in the CE population from whom at least one bacterial pathogen was isolated from blood or infected tissue at baseline. For evaluating safety, the ITT population that included all patients who received any amount of intravenous study drug was used.

### 2.2. Definitions and Outcomes

The primary outcome was overall clinical cure with the resolution of clinical signs and symptoms of cSSSI, or improvement to the extent that no further antimicrobial therapy was necessary at the test-of-cure visit (8–15 days after the last dose of study treatment). Secondary outcomes included the clinical failure rate, microbiologic eradication, relapse rate, and the risk of AEs, including treatment-emergent AEs (TEAEs), serious AEs, and discontinuation because of AEs.

### 2.3. Data Analysis

This study used the Cochrane Risk of Bias Assessment tool to assess the quality of enrolled randomized controlled trials (RCTs) and the risk of bias [21]. Statistical analyses were conducted using the software Review Manager, version 5.3. The degree of heterogeneity was evaluated with the Q statistic generated from the χ^2^ test. The proportion of statistical heterogeneity was assessed using the *I*^2^ measure. Heterogeneity was considered significant when the *p* was <0.10 or *I*^2^ was >50%. The *z*-statistics are significance tests for the weighted average effect size. We use *df* to describe the percentage of the variability in effect estimates that is due to heterogeneity rather than sampling error. Weight is the inverse of the variance of the effect estimate. The random-effects model was used when data were significantly heterogeneous, and the fixed-effect model was used when data were homogenous. Pooled risk ratios (RRs) and 95% confidence intervals (CIs) were calculated for outcome analyses.

## 3. Results

### 3.1. Study Selection and Characteristics

**Figure 1:**
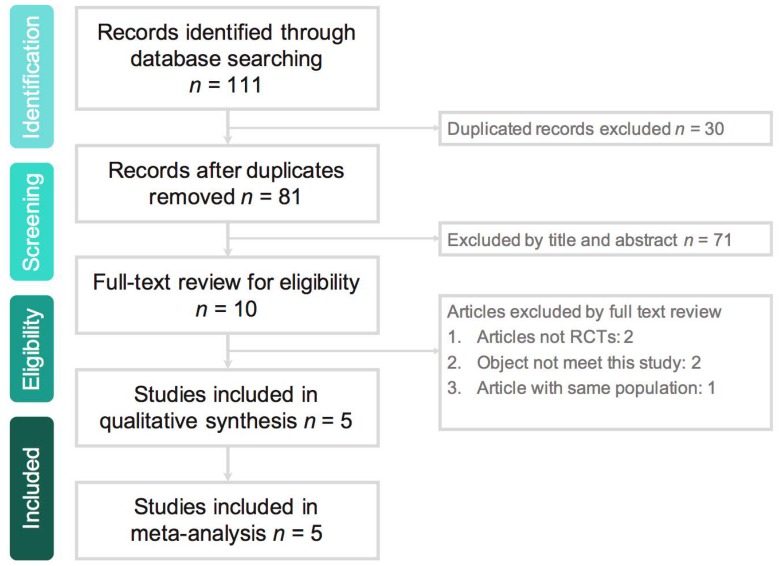
Study selection process flow. RCT: randomized controlled trial.

**Figure 2:**
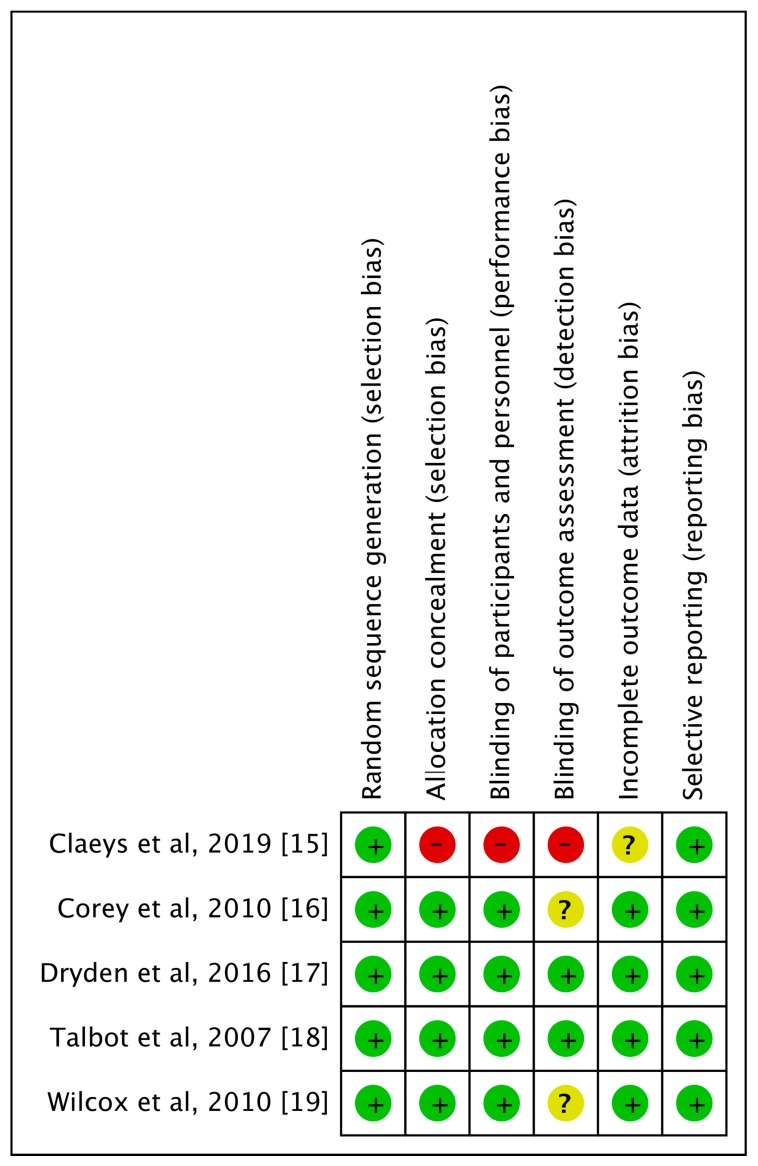
Risk of bias per study and domain.

The search program yielded 111 references. After excluding 30 duplications, the remaining 81 abstracts were screened. Among them, we retrieved ten articles for full-text review. Finally, five studies [15,16,17,18,19] meeting the inclusion criteria were included in this meta-analysis (Figure 1). All studies [15,16,17,18,19] were randomized, multicenter designed to compare the clinical efficacy and safety of ceftaroline with other comparators for adult patients with cSSSIs (Table 1). Overall, a total of 1326 and 1035 patients received ceftaroline and comparators, respectively. All studies [16,17,18,19], except one [15], were multi-national. Four studies [16,17,18,19] compared monotherapy with ceftaroline and vancomycin-based combination therapy. One study [15] used ceftaroline with/without metronidazole in comparison with vancomycin/ceftriaxone/metronidazole combination therapy. Most of the domains in each study were classified as having a low risk of bias, except one study [15] with high risk of selection, performance, and detection bias (Figure 2).

### 3.2. Clinical Efficacy and Microbiologic Response

**Figure 3:**
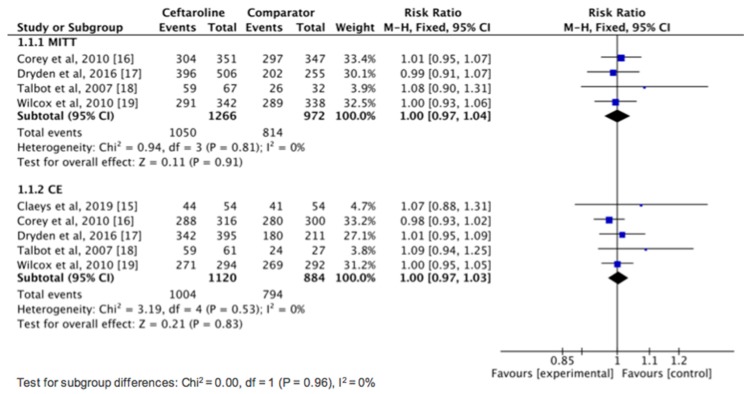
Overall clinical cure rates for ceftaroline and comparators in the treatment of complicated skin and skin structure infections. MITT: modified intention-to-treat; CE: clinically evaluable.

**Figure 4:**
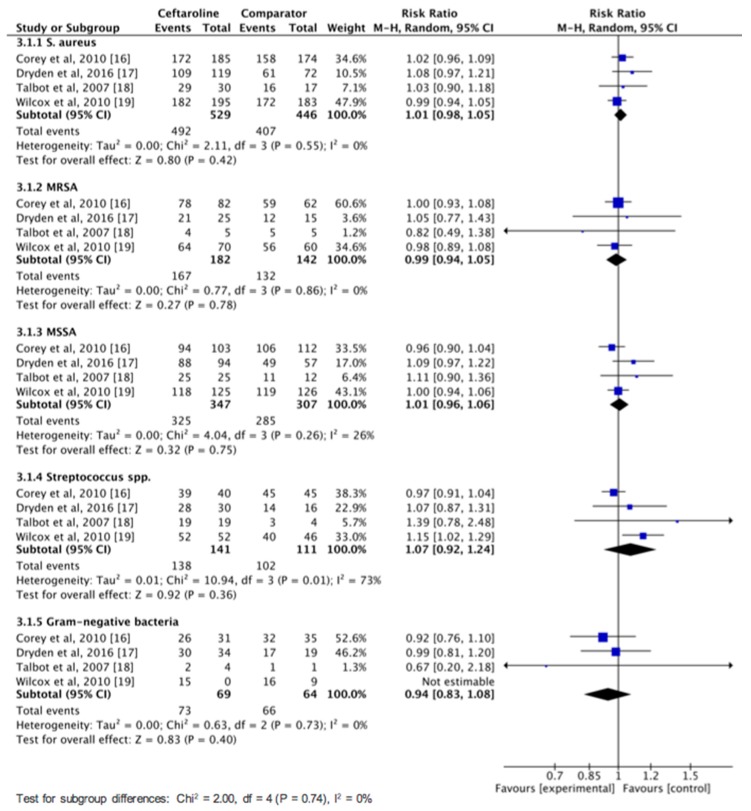
Overall clinical failure rates of ceftaroline and comparators in the treatment of complicated skin and soft tissue infections according to different pathogens. MRSA: methicillin-resistant *Staphylococcus aureus*: MSSA: methicillin-susceptible *S. aureus.*

Overall, ceftaroline had a clinical cure rate in the MITT population that was similar to comparators in the treatment of cSSSIs (82.9% vs. 83.7%, risk ratio (RR), 1.00; 95% CI, 0.97–1.04; *I*^2^ = 0%, Figure 3) in the pool analysis of four studies [16,17,18,19]. In the CE population, no difference was found between ceftaroline and comparators in terms of clinical cure rate in the pool analysis of five studies (89.6% vs. 89.8%, RR, 1.00; 95% CI, 0.97–1.03; *I*^2^ = 0%, Figure 3). In addition, ceftaroline had a lower clinical failure rate in the MITT population than comparators in the treatment of cSSSI (7.4% vs. 11.4%, RR, 0.66; 95% CI, 0.45–0.97; *I*^2^ = 17%). However, in the CE population, no significant difference was observed between ceftaroline and comparator in terms of clinical failure rate (9.3% vs. 7.4%, RR, 1.04; 95% CI, 0.64–1.69; *I*^2^ = 28%). Moreover, we performed a subgroup analysis of clinical cure rate among adult patients with cSSSIs according to different pathogens (Figure 4). No significant difference was found between ceftaroline and comparator for treating infection with *S. aureus* (RR, 1.01; 95% CI, 0.98–1.05; *I*^2^ = 0%), MRSA (RR, 0.99; 95% CI, 0.94–1.05; *I*^2^ = 0%), methicillin-susceptible *S. aureus* (MSSA; RR, 1.01; 95% CI, 0.96–1.06; *I*^2^ = 26%), *Streptococcus* spp. (RR, 1.07; 95% CI, 0.92–1.24; *I*^2^ = 73%), and Gram-negative bacteria (GNB) (RR, 0.94; 95% CI, 0.83–1.08; *I*^2^ = 0%) (Figure 4). Finally, ceftaroline had a similar rate of microbiologic eradication (92.2% vs. 92.6%, RR, 1.00; 95% CI, 0.97–1.03; *I*^2^ = 9%) and relapse (6.9% vs. 9.1%, RR, 0.48; 95% CI, 0.14–1.74; *I*^2^ = 0%) with comparators in the pool analysis of four studies [16,17,18,19].

### 3.3. AEs

**Figure 5:**
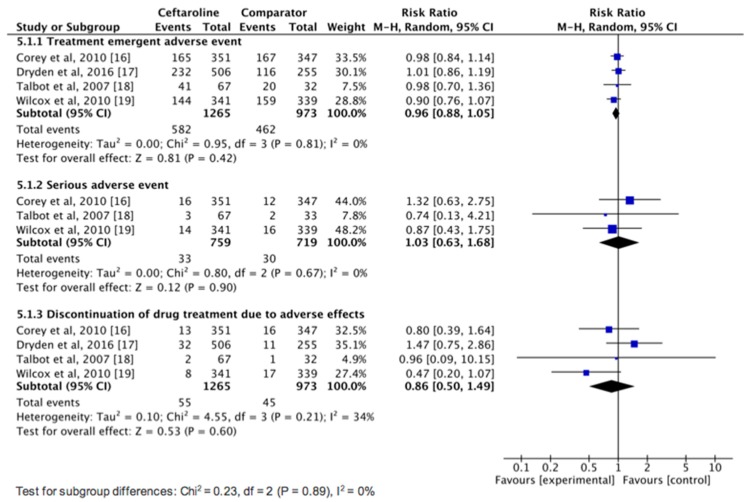
Adverse event risks with ceftaroline and comparators in the treatment of complicated skin and soft tissue infections.

No significant differences were found between ceftaroline and comparators for the risk of TEAEs (RR, 0.96; 95% CI, 0.88–1.05; *I*^2^ = 0%), serious AEs (RR, 1.03; 95% CI, 0.63–1.68; *I*^2^ = 0%), and discontinuation of study drug due to an AE (RR, 0.86; 95% CI, 0.50–1.49; *I*^2^ = 34%) (Figure 5). Regarding common AEs, no significant difference was observed between ceftaroline and comparators in terms of nausea (5.1% vs. 4.7%, RR, 1.13; 95% CI, 0.78–1.64; *I*^2^ = 0%), headache (4.5% vs. 4.6%, RR, 1.00; 95% CI, 0.68–1.46; *I*^2^ = 0%), and diarrhea (3.8% vs. 3.3%, RR, 1.28; 95% CI, 0.82–2.01; *I*^2^ = 0%).

## 4. Discussion

This meta-analysis based on five RCTs found that the clinical efficacy of ceftaroline is similar to that of other comparators in the treatment of adult patients with cSSSIs. This significant finding is supported by the following aspects of the analysis. First, the overall pooled clinical cure rate of ceftaroline in treating acute bacterial infections was 82.9% in the MITT population and 89.6% in the CE population, and it was as good as vancomycin-based combination (83.7% in the MITT population and 89.8% in the CE population). These findings are consistent with a previous meta-analysis [22] of three RCTs, which revealed that no statistically significant difference exists in terms of clinical cure between ceftaroline and vancomycin plus aztreonam (RR, 1.01; 95% CI, 0.97–1.05; *I*^2^ = 0%). Second, the pooled clinical failure rate of ceftaroline in this meta-analysis was only 7.4%, which was lower than comparators in the MITT population. In the CE population, the clinical failure rate was 9.3%, which was similar to comparators. Third, this similarity in terms of clinical efficacy between ceftaroline and comparators did not change with different pathogens. Among each subgroup analysis of cSSSI caused by *S. aureus*, MRSA, MSSA, *Streptococcus* spp., and GNB, ceftaroline exerted similar clinical cure rates as the comparators. Our finding about MRSA is also consistent with a previous meta-analysis [22] of three RCTs that reported no difference in clinical cure outcomes between the subgroups of patients with and without MRSA (test for interaction *p* = 0.52). Finally, we found that ceftaroline had a similar microbiologic eradication rate and relapse rate to comparators in terms of microbiologic response. In summary, all these findings indicated that ceftaroline can be an effective therapeutic option in the treatment of adult patients with cSSSIs.

The effectiveness of ceftaroline in the treatment of cSSSIs in adult patients can be supported by in vitro studies [11,23,24]. In the 7 years of a ceftaroline surveillance program from 2010 to 2016, ceftaroline displayed potent activity against *S. aureus*, including MRSA (with the susceptibility rate of 97.2%) [23]. For *S. aureus* isolated from surgical skin and skin structure infections, ceftaroline was active against all MSSA (minimal inhibitory concentration (MIC)_90_, 0.25 mcg/mL) and nearly all MRSA (MIC_90_, 1 mcg/mL) [11]. Even for MRSA isolates causing bloodstream infection, ceftaroline was active against 95.4% and 100.0% of the isolates at ≤1 and ≤2 mg/L, respectively [24]. Similar findings were observed in several studies [16,17,19] of this meta-analysis. In the study by Corey et al. [16], the MIC_90_ of ceftaroline against MSSA, MRSA, *Streptococcus pyogenes*, and *S. agalactiae* was only 0.25, 1, ≤0.004, and 0.015 mg/L, respectively. In a study by Dryden et al. [17], the MIC_90_ of ceftaroline against MSSA, MRSA, *S. pyogenes*, and *S. agalactiae* was 0.25, 0.5, ≤0.008, and 0.015 mg/L, respectively. In a study by Wilcox et al. [19], the MIC_90_ of ceftaroline against MSSA, MRSA, and *S. pyogenes* were 0.25, 0.5, and ≤0.004 mg/L, respectively. Overall, the potent in vitro activity of ceftaroline against *Streptococcus* spp. and *S. aureus*, including MRSA, in these studies [11,16,17,19,23,24] largely explains the great in vivo clinical response in this meta-analysis.

In addition to clinical efficacy, we should consider AE risk while prescribing ceftaroline. Nausea, headache, and diarrhea were the most common AEs, and the overall incidence of these AEs ranged from 3% to 5%, comparable with comparators. Moreover, the pooled risks of TEAEs and serious AEs were similar between ceftaroline and comparators. Finally, no significant difference was observed between ceftaroline and comparators in terms of discontinuation of the study drug due to an AE. Therefore, the findings of this meta-analysis suggest that ceftaroline is as safe as other comparators in the treatment of cSSSIs among adult patients.

This study has several limitations. First, only five RCTs using similar inclusion criteria were considered in this meta-analysis. Second, the usefulness of ceftaroline in treating cSSSI was not assessed according to the disease severity. Therefore, the generalization of the findings of this meta-analysis may be limited.

In conclusion, ceftaroline is as good as comparators in terms of efficacy and tolerance in the treatment of cSSSI in adult patients. In addition, with ceftaroline’s broad-spectrum activity, including anti-MRSA, it may be used as monotherapy in adult patients with cSSSI. Overall, ceftaroline is an appropriate option for antibiotic therapy in adult patients with cSSSI.

## Figures and Tables

**Table 1:** Clinical trial summary.

Study, Published Year	Study Design	Study Site	No (Male Ratio, %) of Patients		Mean Age of Patients		Dose Regimen	
			Ceftaroline	Comparator	Ceftaroline	Comparator	Ceftaroline	Comparator
Talbot et al., 2007 [18]	Multicenter, randomized, observe-blinded (2:1)	15 clinical sites in USA, South America, South Africa, Russia	67 (55.2)	33 (59.4)	41.6	44.0	600 mg q12h	Vancomycin 1 g q12h ± aztreonam 1 g q8h
Corey et al., 2010 [16]	Multicenter, randomized, double-blind (1:1)	55 sites in 10 countries	351 (62.7)	347 (62.8)	47.2	49.2	600 mg q12h	Vancomycin 1 g q12h + aztreonam 1 g q12h
Wilcox et al., 2010 [19]	Multicenter, randomized, double-blind (1:1)	56 sites in 12 countries	348 (65.5)	346 (59.5)	47.8	47.5	600 mg q12h	Vancomycin 1 g q12h + aztreonam 1 g q12h
Dryden et al., 2016 [17]	Multicenter, randomized, double-blind (2:1)	111 sites in 28 countries	506 (61.3)	255 (58.0)	52.6	53.6	600 mg q8h	Vancomycin 15 mg/kg q12h + aztreonam 1 g q8h
Claeys et al., 2019 [15]	Multicenter, randomized, double-blind (1:1)	3 sites in USA	54 (NA)	54 (NA)	54.8	48.1	± metronidazole *	Vancomycin ± ceftriaxone ± metronidazole or ampicillin/sulbactam *

* dosed based on renal function or per site protocol; NA: not available.

## References

[1] Edelsberg J., Taneja C., Zervos M., Haque N., Moore C., Reyes K., Oster G. (2009). Trends in US hospital admissions for skin and soft tissue infections. Emerg. Infect. Dis..

[2] Klein E.Y., Mojica N., Jiang W., Cosgrove S.E., Septimus E., Morgan D.J., Laxminarayan R. (2017). Trends in methicillin-resistant *Staphylococcus aureus* hospitalizations in the United States, 2010–2014. Clin. Infect. Dis..

[3] Kaye K.S., Patel D.A., Stephens J.M., Khachatryan A., Patel A., Johnson K. (2015). Rising United States hospital admissions for acute bacterial skin and skin structure infections: Recent trends and economic impact. PLoS ONE.

[4] Stryjewski M.E., Chambers H.F. (2008). Skin and soft-tissue infections caused by community-acquired methicillin-resistant *Staphylococcus aureus*. Clin. Infect. Dis..

[5] Stevens D.L., Bisno A.L., Chambers H.F., Everett E.D., Dellinger P., Goldstein E.J., Wade J.C. (2014). Practice guidelines for the diagnosis and management of skin and soft tissue infections: 2014 update by the Infectious Diseases Society of America. Clin. Infect. Dis..

[6] Jorgenson M.R., DePestel D.D., Carver P.L. (2011). Ceftaroline fosamil: A novel broad-spectrum cephalosporin with activity against methicillin-resistant *Staphylococcus aureus*. Ann. Pharmacother..

[7] Poon H., Chang M.H., Fung H.B. (2012). Ceftaroline fosamil: A cephalosporin with activity against methicillin-resistant *Staphylococcus aureus*. Clin. Ther..

[8] Pfaller M.A., Mendes R.E., Castanheira M., Flamm R.K., Jones R.N., Sader H.S. (2017). Ceftaroline activity tested against bacterial isolates causing community-acquired respiratory tract infections and skin and skin structure infections in pediatric patients from United States hospitals: 2012–2014. Pediatr. Infect. Dis. J..

[9] Rolston K.V.I., Jamal M.A., Nesher L., Shelburne S.A., Raad I., Prince R.A. (2017). In vitro activity of ceftaroline and comparator agents against Gram-positive and Gram-negative clinical isolates from cancer patients. Int. J. Antimicrob. Agents.

[10] Farrell D.J., Castanheira M., Mendes R.E., Sader H.S., Jones R.N. (2012). In vitro activity of ceftaroline against multidrug-resistant *Staphylococcus aureus* and *Streptococcus pneumoniae*: A review of published studies and the AWARE Surveillance Program (2008–2010). Clin. Infect. Dis..

[11] Sader H.S., Farrell D.J., Flamm R.K., Jones R.N. (2016). Antimicrobial Activity of ceftaroline tested against *Staphylococcus aureus* from surgical skin and skin structure infections in US medical centers. Surg. Infect..

[12] Zhong N.S., Sun T., Zhuo C., D’Souza G., Lee S.H., Lan N.H., Melnick D. (2015). Ceftaroline fosamil versus ceftriaxone for the treatment of Asian patients with community-acquired pneumonia: A randomised, controlled, double-blind, phase 3, non-inferiority with nested superiority trial. Lancet Infect. Dis..

[13] File T.M., Low D.E., Eckburg P.B., Talbot G.H., Friedland H.D., Lee J., Pullman J. (2011). FOCUS 1: A randomized, double-blinded, multicentre, Phase III trial of the efficacy and safety of ceftaroline fosamil versus ceftriaxone in community-acquired pneumonia. J. Antimicrob. Chemother..

[14] Low D.E., File T.M., Eckburg P.B., Talbot G.H., David Friedland H., Lee J., Corral J. (2011). FOCUS 2: A randomized, double-blinded, multicentre, Phase III trial of the efficacy and safety of ceftaroline fosamil versus ceftriaxone in community-acquired pneumonia. J. Antimicrob. Chemother..

[15] Claeys K.C., Zasowski E.J., Trinh T.D., Casapao A.M., Pogue J.M., Bhatia N., Sherwin R. (2019). Open-label randomized trial of early clinical outcomes of ceftaroline fosamil versus vancomycin for the treatment of acute bacterial skin and skin structure infections at risk of methicillin-resistant *Staphylococcus aureus*. Infect. Dis. Ther..

[16] Corey G.R., Wilcox M.H., Talbot G.H., Thye D., Friedland D., Baculik T. (2010). CANVAS 1: The first Phase III, randomized, double-blind study evaluating ceftaroline fosamil for the treatment of patients with complicated skin and skin structure infections. J. Antimicrob. Chemother..

[17] Dryden M., Zhang Y., Wilson D., Iaconis J.P., Gonzalez J. (2016). A Phase III, randomized, controlled, non-inferiority trial of ceftaroline fosamil 600 mg every 8 h versus vancomycin plus aztreonam in patients with complicated skin and soft tissue infection with systemic inflammatory response or underlying comorbidities. J. Antimicrob. Chemother..

[18] Talbot G.H., Thye D., Das A., Ge Y. (2007). Phase 2 study of ceftaroline versus standard therapy in treatment of complicated skin and skin structure infections. Antimicrob. Agents Chemother..

[19] Wilcox M.H., Corey G.R., Talbot G.H., Thye D., Friedland D., Baculik T. (2010). CANVAS 2: The second Phase III, randomized, double-blind study evaluating ceftaroline fosamil for the treatment of patients with complicated skin and skin structure infections. J. Antimicrob. Chemother..

[20] Sotgiu G., Aliberti S., Gramegna A., Mantero M., Di Pasquale M., Trogu F., Blasi F. (2018). Efficacy and effectiveness of Ceftaroline Fosamil in patients with pneumonia: A systematic review and meta-analysis. Respir. Res..

[21] Higgins J.P., Altman D.G., Gøtzsche P.C., Jüni P., Moher D., Oxman A.D., Sterne J.A. (2011). The Cochrane Collaboration’s tool for assessing risk of bias in randomised trials. Bmj.

[22] El Hajj M.S., Turgeon R.D., Wilby K.J. (2017). Ceftaroline fosamil for community-acquired pneumonia and skin and skin structure infections: A systematic review. Int. J. Clin. Pharm..

[23] Sader H.S., Mendes R.E., Streit J.M., Flamm R.K. (2017). Antimicrobial susceptibility trends among *Staphylococcus aureus* isolates from U.S. Hospitals: Results from 7 Years of the Ceftaroline (AWARE) surveillance program, 2010 to 2016. Antimicrob. Agents Chemother..

[24] Sader H.S., Farrell D.J., Flamm R.K., Jones R.N. (2015). Activity of ceftaroline and comparator agents tested against *Staphylococcus aureus* from patients with bloodstream infections in US medical centres (2009–13). J. Antimicrob. Chemother..

